# Genetic analysis of EMS-induced *AGO5* mutants affecting seed coat pigmentation in soybean (*Glycine max* L.)

**DOI:** 10.1186/s12870-026-09122-4

**Published:** 2026-06-03

**Authors:** Jinwon Lee, Gyu Tae Park, Junbeom Park, Hyun Jo, Jeong-Dong Lee, Hak Soo Seo, Jong Tae Song

**Affiliations:** 1 Department of Applied Biosciences, Kyungpook National University, Daegu, 41566 Republic of Korea; 2 Upland-Field Machinery Research Center, Kyungpook National University, Daegu, 41566 Republic of Korea; 3 Department of Agriculture, Forestry and Bioresources, Seoul National University, Seoul, 08826 Republic of Korea

**Keywords:** Soybean, Seed coat pigmentation, Post-transcriptional gene silencing, *ARGONAUTE5*, Chalcone synthase, EMS mutagenesis

## Abstract

**Background:**

In soybean, seed coat pigmentation is regulated by post-transcriptional gene silencing (PTGS) of *chalcone synthase* (*CHS*) genes at the *I* locus. Although several components of the PTGS pathway have been implicated in this process, genetic evidence linking specific RNA silencing factors to seed coat pigmentation remains limited.

**Results:**

We identified three independent brown seed coat mutants from an EMS-mutagenized population of the soybean cultivar Pungsannamul, which normally produces yellow seeds. Genetic segregation analysis demonstrated that the brown seed coat phenotype in all three mutants is controlled by a single recessive locus. Complementation tests revealed that the three mutants are allelic. Genetic mapping by bulk segregant analysis (BSA) using a SNP genotyping array localized the causal locus to a 2.83-Mb interval on chromosome 11 containing the *ARGONAUTE5* (*AGO5*) gene. Sequence analysis identified distinct mutations in *AGO5* in each mutant line, including a splice-site mutation and two missense substitutions within the conserved P-element Induced Wimpy Testis (PIWI) domain. Co-segregation analysis using derived cleaved amplified polymorphic sequence (dCAPS) markers confirmed a complete association between *AGO5* mutant alleles and the brown seed coat phenotype. Expression analysis revealed significantly elevated *CHS7* transcript levels in the seed coats of all three *AGO5* mutants compared with the wild type.

**Conclusions:**

Our results provide genetic evidence that AGO5 plays a critical role in PTGS-mediated regulation of *CHS* expression and seed coat pigmentation in soybean, consistent with phenotypes of previously reported PTGS component mutants such as *dicer-like2* (*dcl2a/dcl2b*) double mutants.

**Supplementary Information:**

The online version contains supplementary material available at 10.1186/s12870-026-09122-4.

## Background

Soybean (*Glycine max* L.) is an economically and nutritionally important crop and a major source of protein and oil for both human and animal consumption [1]. In addition to providing a physical barrier, the seed coat contains biologically active compounds such as dietary fiber, polyphenols, and flavonoids [2].

Seed coat color is an important agronomic trait in soybean, influencing both market preference and seed quality [3]. Yellow soybeans are commercially preferred for food processing applications, while black and brown soybeans, which accumulate higher anthocyanin levels, offer enhanced antioxidant properties and greater abiotic stress tolerance [3, 4]. Pigmentation of the seed coat and floral tissues is primarily determined by the accumulation of anthocyanins and related flavonoid compounds, which are synthesized through a conserved biosynthetic pathway involving multiple enzymatic steps [5–11].

In soybean, seed coat pigmentation is genetically controlled by the *I* locus on chromosome 8, which comprises at least four alleles (*I*,* i*^*i*^, *i*^*k*^, and *i*, with that order of dominance) encoding a cluster of *chalcone synthase* (*CHS*) genes that catalyze the first committed step of flavonoid and anthocyanin biosynthesis [12, 13]. The dominant *I* allele produces a completely non-pigmented seed coat, whereas *i*^*i*^ restricts pigmentation to the hilum, *i*^*k*^ to a saddle-shaped region, and the recessive *i* allele results in a fully pigmented seed coat [14, 15]. The spontaneous mutation from *I*, or *i*^*i*^ to *i* produces black (*iR*) or brown (*ir*) seed coat colors depending on the *R* locus genotype located on chromosome 9. The *R* locus encodes an R2R3-MYB transcription factor that regulates expression of *UDP-glucose: flavonoid 3-O-glucosyltransferase*, the final enzyme in the anthocyanin biosynthetic pathway, thereby influencing brown hilum or brown seed coat phenotypes [6, 8].

Post-transcriptional gene silencing (PTGS), also known as RNA interference (RNAi), is a conserved small RNA–mediated gene regulatory pathway that represses gene expression through sequence-specific degradation of target transcripts. The pathway is initiated by exogenous or endogenous double-stranded RNA (dsRNA), which is recognized and cleaved by the ribonuclease Dicer-like (DCL) proteins into 20–25 nucleotide small interfering RNAs (siRNAs). These siRNAs are subsequently incorporated as single strands into the RNA-induced silencing complex (RISC), a multiprotein complex containing ARGONAUTE (AGO) proteins. The guide strand within RISC directs sequence-specific cleavage of complementary target mRNAs by the AGO endonuclease, thereby silencing gene expression [16–19].

In plants, PTGS plays critical roles in antiviral defense, transposon silencing, and regulation of endogenous genes involved in development and metabolism [20–22]. At the *I* locus, an inverted-repeat cluster of *CHS* genes triggers endogenous PTGS of *CHS7* and *CHS8* transcripts, resulting in reduced flavonoid accumulation and the characteristic yellow seed coat of cultivated soybean [23–27]. Disruption of this silencing pathway leads to derepression of *CHS* expression and pigmentation, as observed in PTGS-defective mutants such as *dicer-like2* (*dcl2a/dcl2b*) double mutants, and has been proposed to involve the *K* locus on chromosome 11, which encodes *ARGONAUTE5* (*AGO5*) [28, 29].

Despite advances in soybean genomics [30, 31] and the identification of multiple seed color loci [32, 33], studies that directly connect visible pigmentation phenotypes to specific RNAi pathway genes through forward genetic approaches remain scarce. In particular, the identification and characterization of ethyl methanesulfonate (EMS)-induced mutations in RNA silencing components provide valuable genetic resources for dissecting the functional roles of PTGS in regulating metabolic pathways. To provide a clear conceptual framework, this regulatory system can be viewed as a hierarchical PTGS model consisting of three levels: (1) initiation via endogenous dsRNA production at the *I* locus, (2) processing of these dsRNAs into functional siRNAs by Dicer-like enzymes such as *DCL2*, and (3) execution of *CHS* mRNA cleavage by the RISC complex. Although upstream initiation and processing factors have been partially characterized, the precise functional role of the downstream effector, particularly in different genetic backgrounds, remains to be fully elucidated.

In this study, we focused on the execution level of this regulatory hierarchy by characterizing three independent brown seed coat mutants from an EMS-mutagenized soybean population. Through genetic mapping, complementation and co-segregation analyses, and expression analysis of *CHS7*, we demonstrate that mutations in *AGO5* impair PTGS-mediated repression of *CHS* expression, leading to seed coat pigmentation. These non-transgenic alleles provide valuable resources for studying RNA silencing and soybean breeding.

## Materials and methods

### Plant materials

Seeds of the soybean (*Glycine max* L.) cultivar Pungsannamul, which carries dominant *I* and *K1* alleles and a recessive *r* allele harboring an AGGT→AGtt splice-site mutation in the *R* gene and thus produces yellow seed coats with a clear hilum (hereafter referred to as wild type [WT]), were treated with 0.3% EMS to generate an EMS-induced mutant population comprising 3,774 lines [6, 34], from which seed coat color mutants were screened and three lines (PE63, PE407, and PE631) showing a brown seed coat were isolated. To obtain segregation populations, WT was crossed with each of the mutants PE63, PE407, and PE631. Complementation tests were performed among PE63, PE407, and PE631 to determine whether the brown seed coat phenotype was caused by mutations in the same gene. In addition, genetic mapping by bulk segregant analysis (BSA) was performed using segregation populations derived from F₃ lines obtained from a cross between the cultivar Jinpung and PE631. All the experimental populations were grown in the experimental fields at Kyungpook National University (Gunwi, 36°07′N, 128°38′E, Republic of Korea).

### Identification and sequence analysis of the *AGO5* gene via BSA

A physical map was constructed from a cross between the soybean cultivar Jinpung and PE631 using the Affymetrix Axiom^®^ 180 K SoyaSNP genotyping array (Wm82.a2.v1) (Affymetrix, Waltham, MA, USA). Among 113 F₃ seeds, 29 individuals exhibiting clear and stable brown seed coat phenotypes were selected for SNP genotyping, while individuals showing ambiguous or intermediate pigmentation patterns were excluded from the analysis. For BSA, genomic DNA from 20 selected F₃ mutants was pooled to form a bulk sample, whereas the remaining nine F₃ mutants were analyzed individually to identify the locus associated with brown seed coat color. The candidate region containing *AGO5* was delineated by detecting informative SNPs in F_3_ individuals using Microsoft Excel. To identify candidate genes, next-generation sequencing (NGS) of genomic DNA from the mutant and wild type was performed on the Illumina HiSeq™ 2500 platform (Macrogen, Seoul, Korea). Clean reads were aligned to the soybean reference genome Glycine max v2.0 (assembly: GCA_000004515.3) obtained from NCBI, and alignment files were generated in BAM format. For gene structure annotation and positional reference throughout this study, the Phytozome soybean genome assembly Wm82.a2.v1 was used unless otherwise stated. The coding regions of the AGO5 locus were amplified by PCR in Jinpung and three mutants (PE407, PE63, and PE631), and PCR amplicons were sequenced (Solgent, Daejeon, Korea).

### Development of dCAPS markers for *AGO5*

For segregation and co-segregation analysis between the brown seed coat phenotype and the *AGO5* genotype, genomic DNA was extracted from leaf tissues of individual F₂ plants derived from crosses between WT and the PE63, PE407, and PE631 mutant lines. The genotypes of F₂ plants were determined using derived cleaved amplified polymorphic sequence (dCAPS) markers. Seed coat color phenotypes were subsequently evaluated in F₃ seeds harvested from the corresponding F₂ plants. For all three mutants (PE63, PE407, and PE631), a single nucleotide substitution was identified in the *AGO5* gene, and dCAPS markers were designed accordingly. For PE63, a BsrGI restriction site was introduced so that the mutant allele was specifically digested. For PE407, a HaeIII site was created to allow digestion of the mutant allele, whereas for PE631, a FokI site was designed to specifically digest the wild-type allele. PCR amplification was performed for each genotype, and the resulting products were digested with the corresponding restriction enzymes and separated by electrophoresis on a 2% agarose gel. Primer sequences used for dCAPS marker analysis are listed in Table S1.

### RNA extraction and quantitative reverse transcription PCR (qRT-PCR) analysis

Total RNA was extracted from developing seeds collected at 50 days after flowering (DAF) using phenol–chloroform extraction followed by lithium chloride precipitation [35, 36]. For cDNA synthesis, RNA was treated with DNase I, and 2 µg of total RNA, reverse transcriptase (Solgent, Daejeon, Korea), 10 ng of oligo (dT) primers, and 2.5 mM dNTPs were used. The synthesized cDNAs were used as templates for qRT-PCR, which was performed with WizPure™ qPCR Master (SYBR; Wizbiosolutions, Seongnam, Korea) on a LightCycler^®^ 480 real-time PCR system (Roche, Mannheim, Germany). Relative expression levels of the *CHS7* gene were quantified by qRT-PCR. Expression values were normalized using *Cons7* (*Glyma.03G137100*) as an internal reference gene, which has been validated for high stability across soybean tissues [37, 38]. Relative expression levels were calculated using the2^−ΔΔCt^ method, with the expression in the WT set to 1. Data represent the mean ± SD of three biological replicates (*n* = 3). Statistical significance was evaluated using Student’s *t*-test.

### 3′-rapid amplification of cDNA ends (RACE) analysis

3′-RACE PCR was performed as described previously [39]. cDNA was synthesized using a 3′-RACE adapter primer (5′-GTAATACGACTCACTATAGGGC(T)₁₈-3′) and a gene-specific primer. Nested PCR was then carried out with gene-specific primers according to the manufacturer’s instructions, and the amplicons were sequenced (Solgent, Daejeon, Korea). All primers used for 3′-RACE are listed in Table S1.

### Extraction of genomic DNA

Genomic DNA from WT, Jinpung, and brown seed coat mutant lines was extracted for BSA, sequencing, and cosegregation analyses. For BSA, genomic DNA from F₃ individual mutants was isolated using the HiGene™ Genomic DNA Prep Kit (Biofact, Daejeon, Korea).

### Multiple alignment of AGO proteins

All AGO protein sequences were retrieved from the UniProt database, except for the structural reference sequence of *Arabidopsis thaliana* AGO10, which was obtained from the Protein Data Bank (PDB ID: 7SVA). Sequences from diverse plant and non-plant lineages were selected to represent major evolutionary groups: legumes (*Glycine soja*, A0A445I2S2; *Phaseolus vulgaris*, V7AFN4; *Vigna radiata*, A0A1S3TL13; *Medicago truncatula*, A0A072UVR4; *Lupinus angustifolius*, A0A4P1RDX0), other dicot plants (*Brassica oleracea*, A0A0D3BUB9; *Citrus unshiu*, A0A2H5N149; *Coffea canephora*, A0A068UET0; *Nicotiana tabacum*, A0A1S4BAX8; *Populus alba*, A0A4U5LSM5; *Prunus persica*, A0A251NNU3; *Vitis vinifera*, D7SN77; *Cephalotus follicularis*, A0A1Q3CCS4), monocots (*Oryza sativa*, Q851R2; *Triticum turgidum*, A0A446URJ2; *Zea mays*, A0A1D6E2 × 4), a green alga (*Coccomyxa subellipsoidea*, I0YVB2), and representative animals (*Homo sapiens*, Q9H9G7; *Drosophila melanogaster*, Q7KY08; *Caenorhabditis elegans*, Q3LTR7). Multiple sequence alignment was performed using ClustalW (https://www.genome.jp/tools-bin/clustalw), and the resulting alignment was used for comparative motif and conservation analyses.

### Protein structure prediction and structural analysis

Protein structures of soybean AGO5 WT and SNP variants (G627R and A911T) were predicted using AlphaFold2 (ColabFold v1.5.5: AlphaFold2 using MMseqs2) with default parameters. Five models were generated for each sequence, and model 3, which showed the highest average pLDDT score, was selected for further analysis. The predicted structures (unrelaxed models) were superimposed onto the cryo-EM structure of the Arabidopsis AGO–guide RNA complex (PDB: 7SVA). Structural alignments, distance measurements, and local Cα RMSD calculations were performed in PyMOL (https://www.pymol.org/.)

## Results

### Identification of brown seed coat mutants from an EMS-mutagenized population

**Fig. 1:**
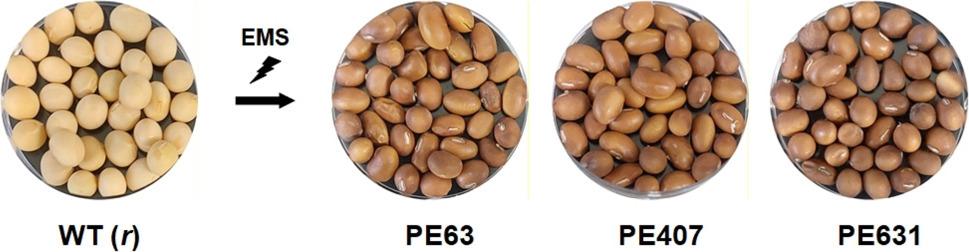
Identification of brown seed coat mutants induced by EMS mutagenesis. Phenotypes of the wild type (WT) and three independent brown seed coat mutants (PE63, PE407, and PE631) generated via EMS treatment of the WT soybean

To obtain genetic materials for validating genes involved in PTGS–mediated regulation of soybean seed coat pigmentation, we screened an EMS-induced mutant pool derived from the soybean cultivar Pungsannamul (WT) for altered seed coat color. While the WT produced seeds with a uniform yellow seed coat, we identified three independent mutant lines (PE63, PE407, PE631) that consistently exhibited a brown seed coat phenotype at maturity (Fig. 1). The pigmentation pattern was clearly distinguishable from that of the WT and was observed across multiple plants within each mutant line, suggesting a genetically controlled trait. Accordingly, we performed genetic segregation analysis to determine the inheritance pattern of the brown seed coat phenotype.

### Genetic segregation analysis of brown seed coat mutants

To examine the inheritance pattern of the brown seed coat phenotype, each mutant line (PE63, PE407, and PE631) was crossed with the WT. F_2_ seeds were obtained from each cross, and segregation of seed coat color was analyzed in the F_3_ generation. In the PE63 × WT population, the observed segregation was 141 yellow to 49 brown seeds, which was consistent with the expected 3:1 ratio (Table 1). Similarly, the PE407 × WT population showed 105 yellow and 33 brown seeds, and the PE631 × WT population showed 68 yellow and 23 brown seeds, both fitting the expected segregation ratio for a single recessive locus (Table 1). Collectively, these results indicate that the brown seed coat phenotype in all three mutant-derived populations is controlled by a single recessive gene.

**Table 1:** Phenotypic segregation of F_3_ seeds and genotypic co-segregation analysis of F_2_ plants at the *AGO5* locus

Parents		Generation	Number of plants	Observed (expected) phenotype		χ^2^ value	*p* value	Observed (expected) genotype			χ^2^ value	*p* value
♀	♂	(seeds)		Yellow	Brown			WT	Hetero	Mutant		
Cross 1^a^												
PE63	P1	F_1_	3		3 (3)							
		F_2_	300	300								
		F_3_	190	141 (142.5)	49 (47.5)	0.06	0.8	47 (48)	94(95)	49 (48)	0.06	0.97
Cross 2^a^												
PE407	P1	F_1_	6		6 (6)							
		F_2_	300	300								
		F_3_	138	105 (103.5)	33 (34.5)	0.09	0.77	29 (31)	63 (63)	33 (31)	0.26	0.87
Cross 3^a^												
PE631	P1	F_1_	5		5 (5)							
		F_2_	300	300								
		F_3_	91	68	23	0.01	0.95	21 (23)	47 (46)	23 (23)	0.19	0.9
Cross 4^b^												
Jinpung	PE631	F_1_	5	5 (5)								
		F_2_	113	113								
		F_3_	113	84 (85)	29 (28)	0.03	0.87					

CAPS marker analysis was performed on F₂ genomic DNA to determine *AGO5* genotypes (Fig. 3). *p* ≥ 0.87 (not significant)

^a^Crosses 1, 2, and 3 were used for genotyping (Fig. 3), P1; Pungsannamul

**Fig. 2:**
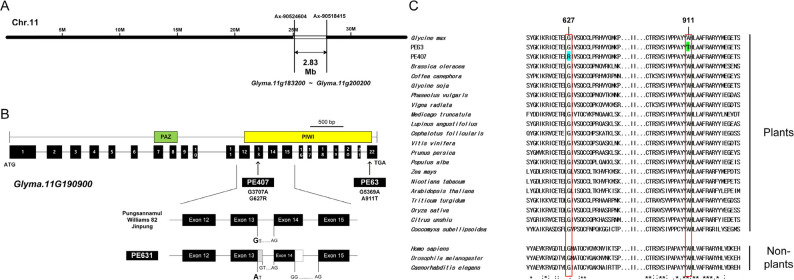
Physical map and gene structure of *AGO5* (*Glyma.11G190900*) in soybean. **A** Physical map of the brown seed coat locus on chromosome 11. A 2.83 Mb candidate region was identified by Affymetrix Axiom^®^ 180 K SoyaSNP genotyping array analysis using pooled genomic DNA from 20 F₃ mutants and individual genotyping of the remaining 9 F₃ mutants derived from a Jinpung × PE631 cross. **B** Gene structure of *Glyma.11G190900* (*AGO5*) identified in this study (consistent with Wm82.a6.v1). Black boxes indicate coding exons (22 total); lines represent introns. Mutation positions and corresponding amino acid changes are marked for PE63 (G5369A, A911T) and PE407 (G3707A, G627R). For PE631, a GT→AT transition at the intron 13 donor site is shown, which triggers aberrant splicing through a cryptic GG donor site. **C** Multiple amino acid alignment of AGO proteins. Sequences from 22 AGOs across plant and non-plant species were aligned with soybean AGO5 (WT, PE63, and PE407). Mutant residues are highlighted in green for PE63 and in cyan for PE407 (both enclosed in red boxes). Asterisks indicate identical residues; colons and periods denote conservative substitutions

^b^Cross 4 was used for physical map construction (Fig. 2). *p* ≥ 0.77 (not significant)

### Complementation analysis of brown seed coat mutants

To determine whether the three brown seed coat mutants were caused by mutations in the same gene or in different genes, we performed complementation analysis among the mutant lines. In crosses between PE407 and PE631, all F₂ seeds (*n* = 200) exhibited the brown seed coat phenotype, and the same phenotype was consistently observed in all F₃ progeny examined (*n* = 64). Similarly, in crosses between PE631 and PE63, all F₂ seeds (*n* = 200) and all F₃ seeds (*n* = 84) displayed the brown seed coat phenotype. The absence of phenotypic complementation in these crosses indicates that the three mutants carry allelic mutations affecting the same genetic locus (Table 2).

**Table 2:** Complementation analysis of brown seed coat mutants showing complete allelism

Parents		Generation (seeds)	Number of plants	Observed phenotype	
♀	♂			Yellow	Brown
Cross 1					
PE407	PE631	F_1_	1		1
		F_2_	200		200
		F_3_	64		64
Cross 2					
PE631	PE63	F_1_	2		2
		F_2_	200		200
		F_3_	84		84

### Gene mapping and identification of *AGO5* gene

To identify the genetic locus underlying the brown seed coat phenotype, we performed BSA using the Affymetrix Axiom^®^ 180 K SoyaSNP Array. A total of 29 F₃ individuals with brown seed coats derived from a cross between the cultivated soybean Jinpung and PE631 were used, in which genomic DNA from 20 individuals was pooled to generate a bulk sample, and the remaining 9 individuals were genotyped separately. The candidate locus was mapped to chromosome 11, flanked by the SNP markers Ax-90,524,604 and Ax-90,518,415, corresponding to an approximately 2.83 Mb interval (Fig. 2A, Table S2).

For consistency in gene annotation, genomic coordinates were interpreted according to the Phytozome soybean reference genome (Wm82.a2.v1), although raw sequencing reads were initially aligned to the NCBI *Glycine max* v2.0 assembly. Based on this annotation framework, the mapped interval spans from.

*Glyma.11g183200* to *Glyma.11g200200* and includes the previously reported *K1* locus encoding *ARGONAUTE5* (*AGO5*; *Glyma.11g190900*) [28]. Sequence analysis of the three brown seed coat mutants (PE63, PE407, and PE631) identified independent single-nucleotide polymorphisms (SNPs) within *Glyma.11g190900*. One variant detected in PE63 was initially annotated within the 3′ untranslated region (UTR) according to Wm82.a2.v1 (Fig. S1A). To clarify the gene structure, we performed 3′ RACE analysis and identified two additional exons downstream of the annotated region, indicating that *Glyma.11g190900* consists of 22 exons (Figs. S1B and S1C). This revised structure is consistent with the updated Phytozome annotation (Wm82.a6.v1) (Figs. 2B and S1B). Based on this refined annotation, the PE63 variant corresponds to a G5369A substitution located within exon 22, resulting in an A911T amino acid change in the C-terminal region of *AGO5* (Figs. 2B and S2).

Sequence analysis of *AGO5* genomic DNA in the PE631 mutant revealed a GT-to-AT transition at the conserved 5′ splice donor site of intron 13. This mutation disrupted canonical splicing, leading to partial retention of intron 13 sequence and a consequent extension of exon 13. In addition, an aberrant splicing event was detected in which a non-canonical GG donor site within exon 14 was spliced to the original AG acceptor site of intron 14. cDNA sequence analysis confirmed that this abnormal splicing causes a frameshift that introduces a premature termination codon in exon 15, thereby truncating the *AGO5* protein (Fig. S3). In PE407, a G3707A substitution resulting in a G627R amino acid change was identified in exon 13. Complementation analysis demonstrated that all three mutants are allelic, confirming that *Glyma.11g190900* underlies the brown seed coat phenotype.

*Glyma.11g190900* encodes an *ARGONAUTE5* (*AGO5*) protein containing the conserved PAZ (Piwi/Argonaute/Zwille) and PIWI (P-element Induced Wimpy Testis domains). The PAZ domain is involved in small RNA binding, whereas the PIWI domain harbors the endonuclease activity essential for RNA interference. Multiple sequence alignment of AGO proteins from diverse organisms revealed that all three amino acid substitutions identified in this study are located within the PIWI domain. Notably, the residues corresponding to G627 and A911 are highly conserved across species (Fig. 2C), suggesting that these mutations may impair *AGO5* function and thereby disrupt PTGS-mediated regulation of seed coat pigmentation.

### Co-segregation of the candidate gene with the brown seed coat phenotype

**Fig. 3:**
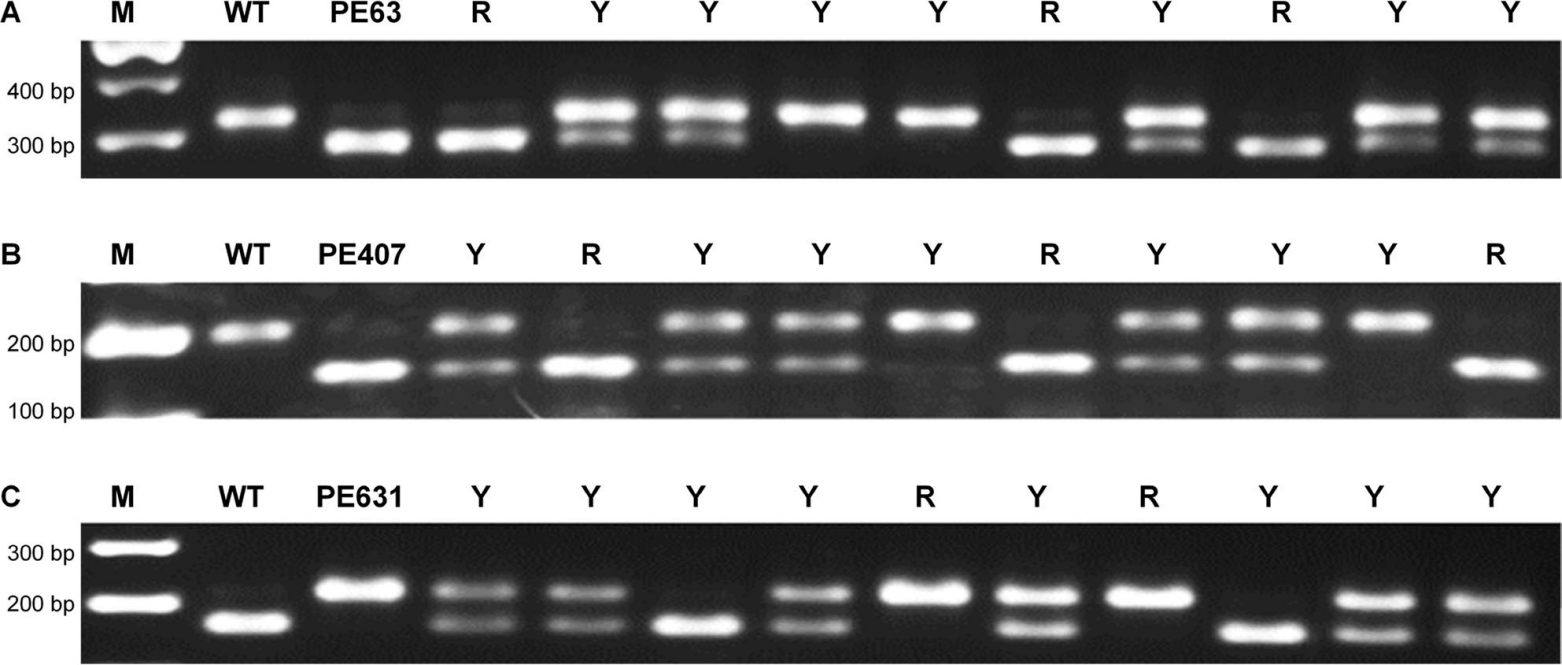
Co-segregation analysis of brown seed coat phenotype with *AGO5* mutations using dCAPS markers. Genomic DNA was extracted from leaves of individual F₂ plants derived from crosses between WT and each mutant line (PE63, PE407, and PE631), followed by dCAPS marker analysis to determine *ago5* genotypes. Seed coat color phenotypes were evaluated in corresponding F₃ seeds. **A** For the PE63 cross, a 209-bp PCR fragment was amplified, with the mutant allele specifically digested by BsrGI. **B** For the PE407 cross, a 206-bp PCR fragment was generated, with the mutant allele digested by HaeIII. **C** For the PE631 cross, a 206-bp PCR fragment was amplified, with the wild-type allele selectively digested by FokI. In all three crosses, dCAPS marker genotypes showed complete co-segregation with the brown seed coat phenotype, confirming that *AGO5* mutations are responsible for the pigmentation defects. M, marker; Y, yellow seed coat; R, brown seed coat

To examine the genetic association between *AGO5* and the brown seed coat phenotype, genomic DNA was extracted from leaves of individual F₂ plants derived from crosses between WT and each mutant line, along with the WT control. dCAPS marker analysis was performed to determine the genotypes of F₂ plants, and seed coat color was subsequently evaluated in F₃ seeds harvested from each corresponding F₂ plant. Analysis of dCAPS marker patterns and seed coat phenotypes revealed complete co-segregation between the *AGO5* mutant alleles and the brown seed coat phenotype in all three crosses (Fig. 3). Segregation analysis based on F₃ seed phenotypes showed that all three crosses followed the expected Mendelian 1:2:1 segregation ratio (wild type: heterozygote : mutant), with no significant deviation as determined by chi-square tests (Table 1). These results indicate that the brown seed coat phenotype is controlled by a single recessive locus and further support the conclusion that the three brown seed coat mutants represent independent alleles of *AGO5*.

### Expression analysis of *chalchone synthase* genes

**Fig. 4:**
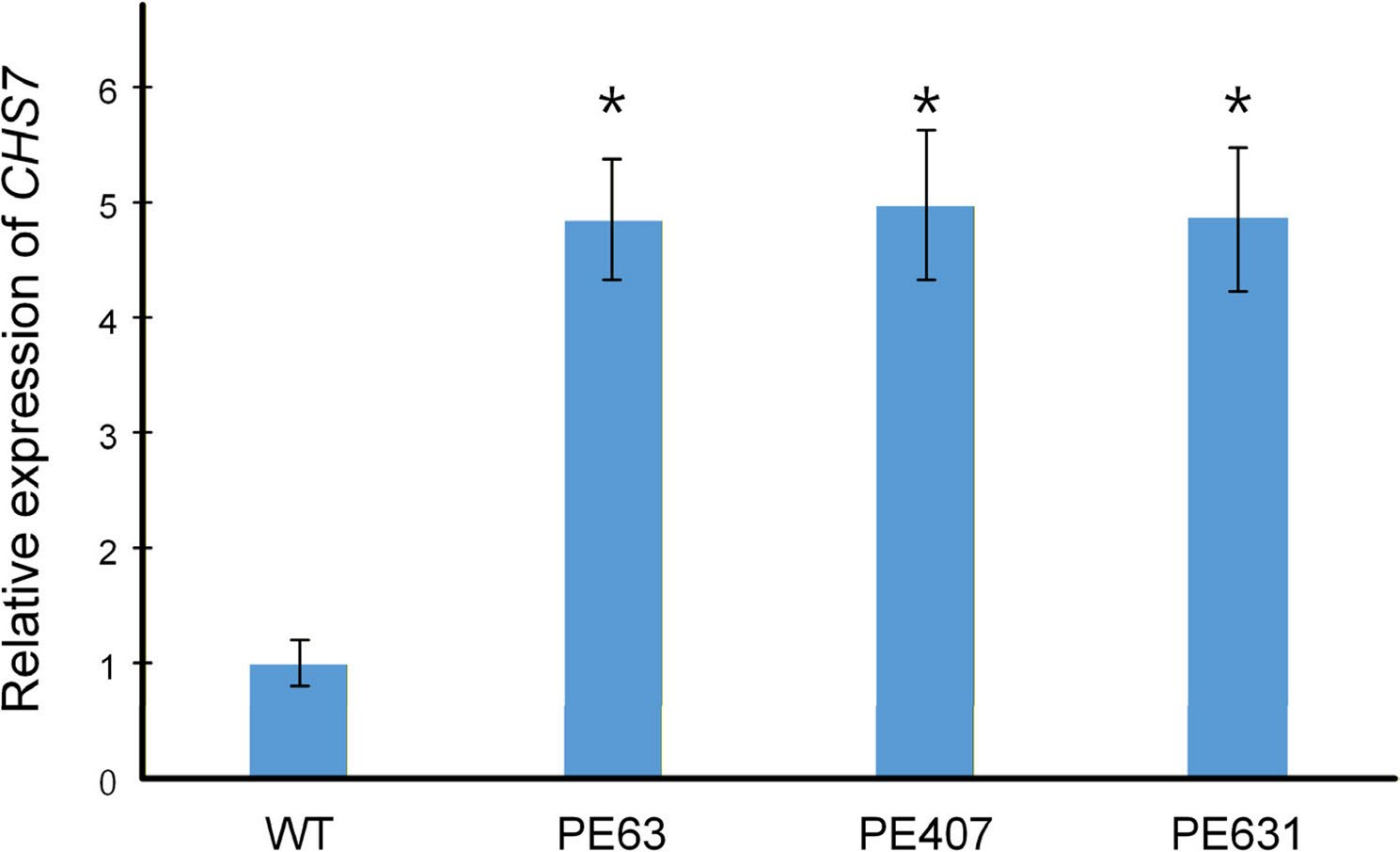
Expression pattern of *CHS7* genes in WT and three brown seed coat mutants. Expression of the *CHS7* gene was analyzed by qRT-PCR in developing seeds collected at 50 DAF from wild-type plants and the PE63, PE407, and PE631 mutants. Error bars represent ± standard deviation of three biological replicates. Expression levels were normalized to constitutive gene (*Cons7*). (*p < 0.001, Student’s *t*-test)

In soybean, pigmentation of flowers and seed coats is associated with the accumulation of anthocyanins, a process regulated by the *I* locus, which contains a cluster of *CHS* genes. Previous studies have shown that the regulation of *CHS* gene expression in the seed coat is mediated by PTGS [23–25, 27]. To examine whether mutations in *AGO5* affect PTGS-mediated regulation of *CHS* genes, we analyzed the expression of *CHS7* in mature seed coats of WT and the three brown seed coat mutants. qRT-PCR analysis revealed that *CHS7* transcript levels were significantly increased in all three *AGO5* mutant lines compared with the wild type (Fig. 4). These results indicate that loss of *AGO5* function compromises PTGS-mediated suppression of *CHS7* expression, leading to increased anthocyanin biosynthesis and the formation of a brown seed coat phenotype. This expression pattern is consistent with previous reports describing elevated *CHS* expression and enhanced seed coat pigmentation in soybean *dcl2a/dcl2b* double mutants defective in PTGS [29].

## Discussion

Seed coat pigmentation in soybean is a complex trait governed by multilayered genetic regulation, primarily involving the flavonoid biosynthetic pathway. In this study, we identified and characterized three independent EMS-induced brown seed coat mutants and demonstrated that all three lesions affect the same locus, *AGO5*, establishing a direct genetic link between *AGO5* function and pigmentation control. Notably, these alleles represent distinct classes of mutations within a single gene: two missense substitutions located in conserved coding regions (PE63 and PE407) and one splice-site mutation (PE631) that induces aberrant transcript processing. The PE631 mutation, which converts the canonical GT donor to AT at intron 13, triggers partial intron retention and activates a cryptic non-canonical donor site, illustrating the flexibility of plant spliceosomal recognition under mutational perturbation [40, 41]. Despite their different molecular origins, all three alleles produced similar brown seed coat pigmentation phenotypes, indicating that disruption of *AGO5* activity is sufficient to derepress pigment accumulation.

The wild type Pungsannamul used for mutagenesis in this study carries dominant *I* and *K1* alleles together with recessive *r*, resulting in yellow seeds. It was previously reported that natural *k1* variants in an *i*^*i*^ background typically produce patterned phenotypes such as saddle pigmentation, whereas loss of *K1* activity in an *I* background results in near-uniform pigmentation [28]. Our EMS-derived *AGO5* mutants phenocopy the loss-of-*K1* phenotype observed in the *I* background, providing genetic evidence that *AGO5* corresponds to *K1*. The distinct pigmentation patterns likely arise from allele-specific silencing domains: whereas *i*^*i*^ restricts silencing to the seed coat excluding the hilum, *I* induces systemic PTGS throughout the tissue. Consequently, *k1* mutations in an *i*^*i*^ background permit pigment leakage into a saddle-shaped domain, while loss of *K1* in an *I* background results in complete failure of the more potent systemic silencing mechanism [28]. Notably, both our *AGO5* mutants and previously reported CRISPR-induced *dcl2a*/*dcl2b* mutants displayed similarly uniform pigmentation [29]. This phenotypic consistency aligns with the hierarchical architecture of the PTGS pathway: DCL2 functions upstream to generate *CHS*-targeting siRNAs, whereas AGO5 acts downstream as an effector that deploys these small RNAs to silence target transcripts. Impairment of siRNA biogenesis at the *DCL2* level or of effector function at the *AGO5* level leads to a breakdown of spatially restricted silencing, thereby enabling pigments to accumulate broadly across the seed coat.

Consistent with this model, expression analysis showed significantly elevated *CHS7* transcript levels in all three *AGO5* mutants. The *I* locus contains an inverted-repeat cluster of *CHS* genes that produces endogenous siRNAs responsible for *CHS* transcript degradation; when core PTGS components are compromised, this silencing system fails, flavonoid biosynthesis proceeds, and pigmented seed coats result. Thus, the transcriptional derepression observed in our mutants provides molecular confirmation that *AGO5* participates directly in the PTGS-mediated repression of *CHS*. While earlier studies identified *K1* as *AGO5* through natural variation [28], our study provides direct functional validation through three independent EMS-induced loss-of-function alleles isolated from a large-scale mutant population of 3,774 lines. The complete co-segregation of these mutations with the pigmentation phenotypes provides definitive genetic evidence that *AGO5* is an essential effector of the PTGS machinery governing soybean seed coat color.

At the molecular level, all three mutations alter conserved residues within the PIWI domain, the catalytic core of AGO proteins. Structural and biochemical studies have shown that Mg²⁺ ions coordinate key residues required for target RNA cleavage [42, 43]. In *Arabidopsis* AGO10, residues corresponding to D709 and H935 participate directly in Mg²⁺-dependent catalysis [44]. Sequence alignment revealed that the A911 residue mutated in PE63 lies immediately adjacent to the conserved catalytic histidine, suggesting that this substitution could influence the local catalytic environment. Although it does not directly alter the catalytic residue itself, it may perturb Mg²⁺ coordination or histidine positioning, thereby reducing cleavage efficiency without disrupting overall RNA binding (Fig. S4).

Supporting this interpretation, structural modeling using AlphaFold, with *Arabidopsis* AGO10 as a reference, revealed no major global structural differences between the wild-type protein and the PE407 or PE63 mutant variants. This suggests that the observed phenotypic effects are unlikely to result from large-scale structural disruptions, but rather from subtle alterations in local residue interactions or protein dynamics that influence *AGO5* catalytic efficiency (Figs. S5 and S6).

Although our findings establish a clear genetic and mechanistic link between *AGO5* dysfunction and PTGS-mediated regulation of pigmentation, further studies will be required to define the precise biochemical consequences of these mutations. In soybean, DCL2 acts upstream of AGO5 to generate *CHS*-targeting siRNAs [29], and previous small-RNA profiling studies have shown that *AGO5* influences their spatial distribution in the seed coat [28]. Detailed biochemical characterization of *AGO5* catalytic activity will therefore be important to determine how the identified mutations quantitatively affect small RNA loading, target cleavage, and silencing efficiency. Nonetheless, our results provide strong genetic evidence that *AGO5* is a key effector of endogenous PTGS controlling flavonoid biosynthesis and seed coat color in soybean.

## Conclusion

In this study, we identified three independent brown seed coat mutants from an EMS-mutagenized soybean population and demonstrated that all three mutations affect the same genetic locus, *ARGONAUTE5* (*AGO5*). Genetic segregation, complementation, and co-segregation analyses collectively confirmed that loss of *AGO5* function is responsible for the brown seed coat phenotype. Molecular characterization revealed distinct mutations in conserved regions of AGO5, and expression analysis showed elevated *CHS7* transcript levels in *AGO5* mutants, consistent with impaired PTGS of *CHS* genes. These results provide genetic support for the role of *AGO5* in regulating seed coat color through RNA silencing pathways in soybean.

## Supplementary Information

Supplementary Material 1. Fig. S1: Genomic structure and 3′ RACE-PCR analysis of the *Glyma.11G190900 (AGO5)* gene. (A) Genomic structure of AGO5 in the Wm82.a2.v1 reference genome. The gene consists of 20 exons. The PE63 mutant site is located within the 3′ UTR. Arrows indicate the positions of the primers (RACE-F1, RACE-F2, RACE-F3, and RACE-R) used for 3′ RACE-PCR. (B) Genomic structure of AGO5 in this study and the Wm82.a6.v1 reference genome. In this version, the gene is composed of 22 exons. Gray boxes indicate two newly identified exons from this study. The PE63 mutant site is located within the exon 22. (C) Agarose gel electrophoresis of 3′ RACE-PCR products. Amplicons generated from the 3′ RACE-PCR are shown. M, DNA size marker. Fig. S2: Full length cDNA sequences of *Glyma.11G190900 (AGO5)* from 3’-RACE PCR. The TGA in red indicates the stop codon in the Wm82.a2.v1 reference sequence, while the TGA in blue corresponds to the stop codon identified in this study, consistent with the Wm82.a6.v1 reference. The green-highlighted A denotes the SNP identified in the PE63 mutant. Fig. S3: Aberrant splicing pattern of the PE631 mutant allele.(A) Schematic representation of the WT genomic DNA and corresponding cDNA sequence spanning exon 13 to exon 15. Exons are shown in uppercase letters, and intron sequences are shown in small capitals. Canonical splice junctions (GT–AG) are indicated in bold. (B) In PE631, a SNP occurs at the intron 13 splice donor site, changing the canonical GT to AT (highlighted in red) and activating a non-canonical donor (GG, highlighted in green) in exon 14. This mutation induces aberrant splicing with partial intron retention and introduces a premature stop codon in exon 15. Underlined nucleotides indicate the GG in exon 14 and the first G of exon 15, which together encode a glycine (G) residue. An asterisk (*) denotes the stop codon. Single-letter codes indicate amino acids. Fig. S4: Sequence alignment of AGO proteins highlighting the PE63 mutation relative to the conserved catalytic histidine. A multiple sequence alignment of AGO proteins from diverse species is shown, including soybean AGO5 WT and mutant variants PE63, Arabidopsis AGO10 used in the slicing-competent AGO–RNA complex (PDB: 7SVA), and representative AGO homologs from other plant species as well as non-plant, totaling 11 AGO proteins. The aspartate residue corresponding to position 686 in soybean AGO5 (D686; corresponding to D790 in Arabidopsis AGO10), which is involved in the catalytic center, is strictly conserved across all analyzed AGO proteins. The position corresponding to the PE63 mutation (A911T in soybean AGO5) is highlighted, together with the adjacent conserved histidine residue (corresponding to H935 in Arabidopsis AGO10), which is a key component of the Mg²⁺-dependent catalytic site. The alignment reveals that the PE63 mutation site is immediately adjacent to this highly conserved catalytic histidine across plant and non-plant AGO proteins, supporting the notion that the PE63 mutation may influence the local catalytic environment or RNA slicing efficiency without directly altering the catalytic residue itself. Fig. S5: Structural modeling of the AGO5 mutation in PE407 (G627R). (A) Front view of the WT *AGO5* structure, highlighting the α-helix (yellow) containing the G627 residue (blue sphere). (B) Structural model of the PE407 mutant, showing the substitution of glycine with arginine at position 627 (R627, red sphere) within the same α-helix context. (C) Merged view showing the superimposition of the WT and PE407 mutant structures, illustrating the steric or electrostatic changes at the C-terminal end of the α-helix. (D) Top view along the helix axis, providing a detailed perspective of the residue 627 orientation at the helix-coil transition. The remaining protein framework is represented in gray. Fig. S6: Structural modeling of the *AGO5* mutation in PE63 (A911T). (A) Front view of the WT AGO5 structure, highlighting the α-helix (residues 905–920, yellow) containing the A911 residue (blue sphere). (B) Structural model of the PE63 mutant, showing the substitution of alanine with threonine at position 911 (T911, red sphere) within the same helical context. (C) Merged view showing the superimposition of the WT and PE63 mutant structures. (D) Top view along the helix axis, illustrating that the helical geometry is largely preserved in the PE63 mutant despite the A911T substitution. The protein backbone and surrounding residues are shown in gray.

Supplementary Material 2. Table S1: Primer lists in this study.

Supplementary Material 3. Table S2: Bulked segregant analysis results using affymetrix axiom® 180K SoyaSNP array data (Wm82.a2.v1).

## Data Availability

The NGS datasets generated and analyzed during the current study are available in the NCBI BioProject repository under accession number PRJNA1429104 (SRA accession number: SRR37384058). The partial coding sequence of AGO5 obtained via 3′ RACE-PCR and the sequence corresponding to the non-canonical splicing variant caused by the splice donor site mutation in PE631 have been deposited in GenBank under accession numbers PZ097986 and PZ097987, respectively. The soybean 180 K SNP array genotyping data generated in this study are provided as an Excel file in the Supplementary Information. All other data supporting the findings of this study are included within this article and its supplementary materials.

## Abbreviations

CHS: Chalcone synthase
PTGS: Post-Transcriptional Gene Silencing
AGO: ARGONAUTE5
dCAPS: Derived Cleaved Amplified Polymorphic Sequence
RNAi: RNA interference
DCL2: Dicer-Like2
EMS: Ethyl Methanesulfonate
qRT-PCR: Quantitative Reverse Transcription PCR
PIWI: P-element Induced Wimpy Testis
PAZ: Piwi/Argonaute/Zwille
siRNAs: Small interfering RNAs
SNP: Single-Nucleotide Polymorphisms
UTR: Untranslated Region
