# Supplementary material for: Genetic analysis of EMS-induced AGO5 mutants affecting seed coat pigmentation in soybean (Glycine max L.)
Source: BMC Plant Biol. 2026 Jun 3;26:1329. doi: 10.1186/s12870-026-09122-4 (PMC13450469; doi:10.1186/s12870-026-09122-4)
Supplement: Supplementary file 1 [file 12870_2026_9122_MOESM1_ESM.docx]

**Fig. S1. Genomic structure and 3′ RACE-PCR analysis of the *Glyma.11G190900* (*AGO5*) gene.**

(A) Genomic structure of *AGO5* in the Wm82.a2.v1 reference genome. The gene consists of 20 exons. The PE63 mutant site is located within the 3′ UTR. Arrows indicate the positions of the primers (RACE-F1, RACE-F2, RACE-F3, and RACE-R) used for 3′ RACE-PCR. (B) Genomic structure of *AGO5* in this study and the Wm82.a6.v1 reference genome. In this version, the gene is composed of 22 exons. Gray boxes indicate two newly identified exons from this study. The PE63 mutant site is located within the exon 22. (C) Agarose gel electrophoresis of 3′ RACE-PCR products. Amplicons generated from the 3′ RACE-PCR are shown. M, DNA size marker.

>***Glyma.11G190900* (*AGO5*)**

ATGTCTCGTCGCGGTGGCTCTAAACAACAACCAGATTCACGCCATCCAGCACCACCGTCTGCGGCGCCTTCTCCCGCCGGAAGAGGACGCGGCCGTGGCCGCGGAGAATTCTCCTCAGTTCCCGCTCCTTCTCCGGTGAATGCACCATCCGCTCCTCCTCCGGCTTCCACCATCGGCGTTCCTTCCGGCGCCGCACCTGTTCGTGCCGCGTCTCCGTCTCCGGCGGCAGAATCTCTCACTTCCGCTGTCGAAAAGCAACTTACATTGCAACCGTCGGCGCCTTCGTCGACGAAGGCGGTGAGGTTCAGTGAACGGCCAGGGTTCGGTCTTGTAGGGAAGAAAATCAAAGTTCGAGCGAATCATTTCCAAGTCAAAGTCGCTGAACAAGATCTATTTCATTATGACGTCTCTATCAATCCGGAGATTAATTCGAAGAAGGTTTGCAGAGATGTTATGACTCTGCTTGTTCAAGCGCACCGTGAAAAGTTTCTTGGAAATCGCATACCGGCCTACGATGGGCGAAAGAGTCTTTTCACCGCCGGACCCTTGCCCTTCGAATCGAAGGATTTTGTGATCGTGCTGAAGGATGAAGATGAACCAGGCTCGTCTTCTTCCGCGCCTGCTAGAAAAAAACGTGAACGCGAGTTTAGGGTCACCATCAGGTTTGCTTCCAGGACTGACCTTCACCATCTCGGGCAGTTTCTCAGGCGTCGTCAGTTGGATTGTCCTTATGAAACTATTCAGGCTCTTGATGTTGTTCTGCGTGCTACTCCATCTGAAAGGTTCGATGTGGTGGGAAGATCGTTTTTCTCGCCTTTTTTGGGGAAACCTGGAACGCTTGGTAGCGGAACGGAGTATTGGAGGGGCTATTACCAGAGCCTTCGTCCAACTCAGATGGGCCTGTCTCTAAATATTGATGTGTCAGCAAGGGCGTTTTATGAAGCTATTCCTGTGATTGATTTCATTCAAATCCATTTTAGGCTCAATCCTTCCAAGCCTCTGCCTGATCAGGATCGAATCAAGCTTAAGAGAGCATTGAGAGGAATCAAGGTAGAAGTTAATCACGGAAAGAATCTTAGACGTTACAAGATCACTGGAGTCACAAAAGAACCACTCAGAGAGTTAATGTTTACTCTTGATGACAAAAGAACAAAAAGCTCAGTTGTTCAATATTTTCATGAGAAATACAATATTGTGTTGAAGCATACACATCTTCCTGCTCTTCAAGCTGGCAGTGACAGTAAACCAATTTTTTTGCCTGTTGAGCTTTGTCAAATTGTTGCTGGACAGAGATATACAAAGAGATTGAATGAGGAGCAAGTAACTAATCTTTTAAGGGCAACTTGTCAGCGTCCTCATGATAGAGAAAACTCTATCAAACAGGTTGTGAAGCAAAGTAATTTCAGCACAGACAAATTTGTGTGTCACTTTGGAATTCAAGTGAAGGAGGAACCAGCATTGCTTGATGCTCGGGTTTTGCCTCCCCCAATGCTAAAATATCATGGCACAGGTAGAGAATCATGTGTTCAACCCAGAACAGGTCAATGGAATATGATTGATAAGAAAATGGTTAATGGTGGCGCTGTGCAGCATTGGACTTGCCTCAACTTTTCTGGAAAAACAAACCGAGGTTTGGCAGCTTCATTTTGTCAAGAGTTGGCCAAAATGTGTAATAACAAGGGCATGCGTTTTAACTTGGATCCTTTGCTGCCCATAACATCTGTTCATAGTAGTCAAGTAGAGAGTGCTCTTGGGAATGTGCATAAGCAGGCTATTGCAAAACTAGCAAACGAAGGAAGACTCGAATTATTGATCATAATTTTGCCTGATTTGAAGGGATCCTATGGGAAAATAAAGCGTATTTGTGAAACTGAGTTAGGGATAGTATCTCAGTGTTGTCTTCCGAGGCATGTTTACCAGATGAAGCCACAATATCTTGAAAATGTGGCCCTCAAGATAAATGTGAAGGTTGGTGGCAGTAACACAGTATTGAATGATGCATTTACTAGAAGAATTCCTCACGTGTCTGACTTACCAACAATAATCTTGGGTGCAGATGTAACACATCCACAGCCAGGGGAAGATTATAGTCCTTCTATTGCTGCAGTAGTTGCATCAATGGATTGGCCTTATGTAACAAAGTACAGAGGAGTTGTTTCTGCTCAAACTCACCGTGAAGAAATCATCCAAGATCTTTATAATACACATGAAGATCCTGTGAGGGGAAAGACACATTCGGGAATTATCAGGGAATTACTTCGTGCTTTCCGTTTATCAACTAAGACTAAGCCAGAGAGGATTATATTCTACAGAGATGGAGTAAGTGAGGGCCAATTCAGCCAGGTTTTGCTGTACGAGATGGATGCAATACGACGCGCTTGTGCCTCACTACAAGAAGACTATATGCCCCGTGTTACTTTTGTGGTGGTCCAGAAAAGACACCACACAAGGTTATTTCCTGCAGAACACGGAAGTCGTGATCAGACAGATAAAAGTGGAAATATATTGCCAGGGACTGTCGTGGACACTCAAATATGCCACCCTCGGGAGTTTGATTTTTACCTCAACAGTCATGCTGGAATACAAGGAACTAGTCGACCAACACATTATCATGTGCTGTTCGATGAAATAACTTCACTGCTGACGAGTTGCAAATGCTTACAAATAACT**TGA**CTTATACGTATGCAAGGTGTACTCGATCAGTCTCAATAGTTCCGCCTGCATATTAT**A**CCTTCAGGGCTCGCTATTACATGGAAGGTGAAACATCAGATTCTGGTTCTGCAAGTGGAGGCCGGACTGCTAACTTTGAGGTTAAATTGCCTTCGGTTAAGGAAAATGTGAAAGATGTGATGGCACATTTGGCTTTCTTCTGT**TGA**

**Fig. S2. Full length cDNA sequences of *Glyma.11G190900* (*AGO5*) from 3’-RACE PCR.**

The TGA in red indicates the stop codon in the Wm82.a2.v1 reference sequence, while the TGA in blue corresponds to the stop codon identified in this study, consistent with the Wm82.a6.v1 reference. The green-highlighted A denotes the SNP identified in the PE63 mutant.

**Fig. S3. Aberrant splicing pattern of the PE631 mutant allele.**
(A) Schematic representation of the WT genomic DNA and corresponding cDNA sequence spanning exon 13 to exon 15. Exons are shown in uppercase letters, and intron sequences are shown in small capitals. Canonical splice junctions (GT–AG) are indicated in bold. (B) In PE631, a SNP occurs at the intron 13 splice donor site, changing the canonical GT to AT (highlighted in red) and activating a non-canonical donor (GG, highlighted in green) in exon 14. This mutation induces aberrant splicing with partial intron retention and introduces a premature stop codon in exon 15. Underlined nucleotides indicate the GG in exon 14 and the first G of exon 15, which together encode a glycine (G) residue. An asterisk (*) denotes the stop codon. Single-letter codes indicate amino acids.

**Fig. S4. Sequence alignment of AGO proteins highlighting the PE63 mutation relative to the conserved catalytic histidine.**

A multiple sequence alignment of AGO proteins from diverse species is shown, including soybean AGO5 WT and mutant variants PE63, *Arabidopsis* AGO10 used in the slicing-competent AGO–RNA complex (PDB: 7SVA), and representative AGO homologs from other plant species as well as non-plant, totaling 11 AGO proteins. The aspartate residue corresponding to position 686 in soybean AGO5 (D686; corresponding to D790 in *Arabidopsis* AGO10), which is involved in the catalytic center, is strictly conserved across all analyzed AGO proteins. The position corresponding to the PE63 mutation (A911T in soybean AGO5) is highlighted, together with the adjacent conserved histidine residue (corresponding to H935 in *Arabidopsis* AGO10), which is a key component of the Mg²⁺-dependent catalytic site. The alignment reveals that the PE63 mutation site is immediately adjacent to this highly conserved catalytic histidine across plant and non-plant AGO proteins, supporting the notion that the PE63 mutation may influence the local catalytic environment or RNA slicing efficiency without directly altering the catalytic residue itself.

**Fig. S5. Structural modeling of the *AGO5* mutation in PE407 (G627R).**

(A) Front view of the WT AGO5 structure, highlighting the α-helix (yellow) containing the G627 residue (blue sphere). (B) Structural model of the PE407 mutant, showing the substitution of glycine with arginine at position 627 (R627, red sphere) within the same α-helix context. (C) Merged view showing the superimposition of the WT and PE407 mutant structures, illustrating the steric or electrostatic changes at the C-terminal end of the α-helix. (D) Top view along the helix axis, providing a detailed perspective of the residue 627 orientation at the helix-coil transition. The remaining protein framework is represented in gray.

**Fig. S6.** **Structural modeling of the *AGO5* mutation in PE63 (A911T).**

(A) Front view of the WT AGO5 structure, highlighting the α-helix (residues 905–920, yellow) containing the A911 residue (blue sphere). (B) Structural model of the PE63 mutant, showing the substitution of alanine with threonine at position 911 (T911, red sphere) within the same helical context. (C) Merged view showing the superimposition of the WT and PE63 mutant structures. (D) Top view along the helix axis, illustrating that the helical geometry is largely preserved in the PE63 mutant despite the A911T substitution. The protein backbone and surrounding residues are shown in gray.
