# Supplementary material for: Genetic analysis of EMS-induced AGO5 mutants affecting seed coat pigmentation in soybean (Glycine max L.)
Source: BMC Plant Biol. 2026 Jun 3;26:1329. doi: 10.1186/s12870-026-09122-4 (PMC13450469; doi:10.1186/s12870-026-09122-4)
Supplement: Supplementary file 2 [file 12870_2026_9122_MOESM2_ESM.docx]

**Table S1. Primer lists in this study**

| **Primer name** | **Primer sequence** | **PCR size** | **Restriction enzyme** | **Restriction site** | **SNP** |
| --- | --- | --- | --- | --- | --- |
| 63815dCAPs-F | AATTTCATTCGGAGTTTATTCTATTTTTGCAGTTCCGCCTGCATATTGT | 209 bp | BsrGI | Mutant | PE63 |
| 63815dCAPs-R | CATACCACAGCCTTCAACAG |  |  |  |  |
| Gmk-F4 | AGCAAACGAAGGAAGACTCGA | 206 bp | HaeIII | Mutant | PE407 |
| 407dCAPs-HaeIII-R | GGCTTCATCTGGTAAACATGCCTCGGAAGACAACACTGAGATACTAGGC |  |  |  |  |
| 631-F-FokI | GATGAAGCCACAATATCTTGAAAATGTGGCCCTCAAGATAAATGTGGAT | 206 bp | FokI | Wild | PE631 |
| dCAPs-R2 | TTGTTGGTAAGTCAGACACG |  |  |  |  |
| GmCHS7-qPCR_F1 | AACCCACCAAACCGTGTTGAT | qRT-PCR | | | |
| GmCHS7-qPCR_R1 | CTTGTCACACATGCGCTGAAAT |  |  |  |  |
| Cons7-F | ATGAATGACGGTTCCCATGTA | qRT-PCR | | | |
| Cons7-R | GGCATTAAGGCAGCTCACTCT |  |  |  |  |
| K-RACE-F | CTGTGAGGGGAAAGACACATTC | 3' RACE PCR | | | |
| K-RACE-F2 | GTGGACACTCAAATATGCCA |  |  |  |  |
| K-RACE-F3 | CAGGGCTCGCTATTACATGG |  |  |  |  |
| Race-R | GAAACCCCTTCCAATTTTCC |  |  |  |  |
